# Evaluation of myocardial glucose metabolism in hypertrophic cardiomyopathy using ^18^F-fluorodeoxyglucose positron emission tomography

**DOI:** 10.1371/journal.pone.0188479

**Published:** 2017-11-27

**Authors:** Rie Aoyama, Hitoshi Takano, Yasuhiro Kobayashi, Mitsunobu Kitamura, Kuniya Asai, Yasuo Amano, Shin-ichiro Kumita, Wataru Shimizu

**Affiliations:** 1 Department of Cardiovascular Medicine, Nippon Medical School, Tokyo, Japan; 2 Department of Radiology, Nippon Medical School, Tokyo, Japan; 3 Department of Radiology, Nihon University Hospital, Tokyo, Japan; University of Louisville, UNITED STATES

## Abstract

**Background:**

The purposes of this study were to assess the usefulness of myocardial ^18^F-fluorodeoxyglucose (^18^F-FDG) positron emission tomography (PET)/computed tomography (CT) for evaluating myocardial metabolic status in hypertrophic cardiomyopathy (HCM) and the therapeutic efficacy of alcohol septal ablation (ASA) in hypertrophic obstructive cardiomyopathy (HOCM).

**Methods:**

Thirty HCM patients (64.4±10.5 years, 14 male, 12 hypertrophic non-obstructive cardiomyopathy [HNCM], 16 HOCM, and 2 dilated phase of HCM) underwent ^18^F-FDG-PET/CT. ^18^F-FDG uptake was semi-quantitatively evaluated using an uptake score in each 17 segment and the entire LV or regional standardized uptake value (SUV).

**Results:**

^18^F-FDG uptake was observed mostly in a hypertrophied myocardium in HNCM patients, whereas ^18^F-FDG was extensively accumulated beyond the hypertrophied myocardium in HOCM patients. There was a positive correlation between the summed uptake score of ^18^F-FDG and high-sensitive troponin T level in HNCM patients (r = 0.603, p = 0.049), whereas the score was positively correlated with brain natriuretic peptide level (r = 0.614, p = 0.011) in HOCM patients. In 10 patients who received ASA, the maximum SUV of the entire LV was significantly reduced from 5.6±2.6 to 3.2±2.1 (p = 0.040) after ASA. Reduction of that maximum SUV was particularly significant in the lateral region (from 5.5±2.6 to 2.9 ±2.2, p = 0.024) but not significant in the anteroseptal region (from 4.5±2.6 to 2.9±1.6, p = 0.12).

**Conclusion:**

Extensive ^18^F-FDG uptake beyond the hypertrophied myocardium was observed in HOCM. ASA attenuates ^18^F-FDG uptake in a remote lateral myocardium.

## Introduction

Hypertrophic cardiomyopathy (HCM) is characterized by asymmetric left ventricular (LV) hypertrophy in the absence of other cardiac or systemic diseases that may cause cardiac hypertrophy [1]. The symptom and clinical course of HCM vary, regardless of the type and severity of HCM [2]. Although many patients stay asymptomatic and experience no serious cardiac event during their life time, some develop sudden death, refractory heart failure, repetitive syncope, and/or severe angina [1]. These various clinical presentations are closely associated with narrowing LV capacity, intra-LV obstruction, severe tissue degradation, extensive myocardial fibrosis, and diastolic dysfunction [1, 3]. Myocardial ischemia also plays an important role in those conditions [4, 5]. The pathohistological degradation involves not only the myocytes but also small coronary arteries, which cause microcirculation disturbance in some HCM patients [6]. Demand ischemia caused by supply/demand mismatch can also occur, particularly when the myocardium contracts against pressure overload [7]. It is important to understand whether myocardial ischemia is responsible for these symptoms when treating symptomatic HCM patients.

The significance of radionuclide imaging with a metabolic tracer in HCM has been reported [8, 9]. These methods are useful, particularly for evaluating myocardial ischemia in HCM, since fatty acid metabolism shifts to glucose metabolism in the setting of ischemia [10, 11]. We therefore focused on regional myocardial energy metabolism in various types of HCM patients using myocardial ^18^F-fluorodeoxyglucose (^18^F-FDG) positron emission tomography (PET)/computed tomography (CT). We sought to determine the relationship between the ^18^F-FDG uptake and type and severity of HCM or clinical parameters in HCM patients. Furthermore, we also assessed whether regional ^18^F-FDG uptake indicating energy metabolic shift or inflammatory response, can be altered by septal reduction therapy with alcohol septal ablation (ASA) in hypertrophic obstructive cardiomyopathy (HOCM) patients suffering from heart failure or angina symptoms refractory to medical treatment.

## Material and methods

### Patient population

This study has been approved by Institutional Review Board in Nippon Medical School, and has been conducted according to the principles expressed in the Declaration of Helsinki. Written informed consent was obtained from the participants. Between April 2013 and May 2016, 36 new patients with HCM were referred to the HCM clinic from other institutions for ASA or further medical treatment. Two patients with a dilated phase of HCM (DHCM) who used to have a typical HCM apparatus and currently had dilated cardiomyopathy-like echocardiographic findings were also included. Among them, 30 patients who agreed to receive ^18^F-FDG-PET/CT were included in this study. The inclusion criteria of this study were 1) the presence of a maximal LV wall thickness ≥15mm by transthoracic echocardiography (TTE) performed before initiation of medical treatment [12], and 2) the absence of other conditions that might explain left ventricular hypertrophy (LVH) during the clinical course. The diagnosis of HCM was based on electrocardiogram (ECG) and echocardiographic demonstrations. ECG and TTE showed a non-dilated, asymmetrical, hypertrophic LV in the absence of other cardiac or systemic diseases that could produce hypertrophy. All patients underwent either coronary angiography or coronary CT angiography and were confirmed to have no significant (>50%) coronary artery disease. Those who had significant valvular heart disease except mitral regurgitation, concomitant neoplasm, or poorly controlled diabetes mellitus (fasting blood sugar level ≥ 120 mg/dL) were not included in this study. HOCM was defined when intra-LV pressure gradient was > 30 mmHg at rest as seen on TTE [13]. A complete history was obtained and clinical examination was performed, along with assessments of New York Heart Association (NYHA) functional class, present drug therapy, and device therapy. We measured serum high-sensitive troponin T (HSTnT) and brain natriuretic peptide (BNP) levels with an AIA-2000ST analyzer (TOSOH, Tokyo, Japan) in accordance with the manufacturer’s instructions. The imaging protocol consisted of TTE, ^18^F-FDG-PET/CT, and cardiac magnetic resonance (CMR) imaging. They received those examinations within 3 weeks. The patients who received ASA underwent repeat imaging protocol approximately 6 months after ASA. Medication was kept constant during the study.

### TTE study

We performed two-dimensional (2D), M-mode, and Doppler echocardiographic studies with PHILIPS IE 33 (Philips Healthcare, Best, The Netherlands) or GE Vivid E 9 ultrasound systems. We measured wall thickness (intraventricular septal thickness [IVST]; posterior wall thickness [PWT]), LV diastolic function (E/A [peak early transmitral filling velocity/peak late transmitral filling velocity], and E/e’ [peak early transmitral filling velocity/peak early diastolic mitral annulus velocity] of the septal side or lateral side on tissue Doppler imaging) and maximum pressure gradient in the LV at rest.

### ^18^F-FDG-PET/CT imaging

^18^F-FDG-PET/CT imaging proceeded as described [14]. Briefly, patients underwent dietary preparation with 24 hours of carbohydrate restriction (glucose < 10g) to suppress the physiological uptake of ^18^F-FDG. 60 min after intravenous administration of a 4MBq/kg dose of ^18^F-FDG, we acquired PET and CT images using a PET/CT scanner with 16-slice CT (GEMINI TF 16, Philips Healthcare, Best, The Netherlands). We checked the blood sugar level before the test to confirm that it is under 120mg/dl. Diabetic patients who received oral hypoglycemic agents or insulin were not included in the present study.

### ^18^F-FDG-PET/CT imaging analysis

We analyzed all PET/CT data as described [14] with a computer workstation. For the visual analysis of PET, we defined the ^18^F-FDG uptake as positive if it was greater than that for a physiologically normal liver. We used the AHA 17-segment model of the LV myocardium to visually localize ^18^F-FDG accumulated myocardium [15] using a semi-quantitative uptake score. Scores were defined as the following; 0: no uptake, 1: slight uptake, 2: mild uptake 3: moderate uptake, and 4: dense uptake. Extent score was calculated as the number of segments with ^18^F-FDG uptake. To compare ^18^F-FDG uptake between pre- and post-ASA, The difference score was calculated by subtracting the post-value from the pre-value for each segment. We also performed another semi-quantitative analysis. 2D regions of interest (ROI) were drawn on the transaxial slices of the PET images to measure the standardized uptake value (SUV) of the entire or regional (anteroseptal, inferior, or lateral) LV myocardium; SUV = (peak kBq/mL in ROI)/(injected activity/g body weight). Three investigators (two radiologists and a cardiologist) separately interpreted ^18^F-FDG-PET/CT findings. Consensus was reached in case of discrepancy.

### CMR imaging

The electrocardiography-gated CMR protocol proceeded with breath-holding as described [16] using an Achieva 1.5 and 3.0 T (Philips Healthcare, Best, The Netherlands). Patients who had a pacemaker, claustrophobia, or renal dysfunction were excluded. Twenty-four patients received the CMR imaging with gadolinium enhancement and one received that without enhancement.

### CMR imaging analysis

We measured the LV myocardial mass index (g/cm2), LV ejection fraction using Simpson’s method, mitral regurgitation (MR) jet, left ventricular outflow tract (LVOT) jet, and left atrial diameter on cine steady state free precession using a workstation (View Forum, Philips, Best, The Netherlands). Additionally, experienced radiologists evaluated imaging of late gadolinium enhancement (LGE) to clarify its presence. We applied the American Heart Association (AHA) 17-segments method to directly compare imaging and ^18^F-FDG-PET/CT for the visual evaluation of the extent of LGE.

### ASA procedure

We performed ASA in 10 hypertrophic obstructive cardiomyopathy (HOCM) patients out of 20 candidates. Indication for HOCM was determined according to the following criteria: 1) symptoms were life limiting after optimization of medication, 2) resting or provoked gradient > 50 mmHg that was confirmed by at least one method during simultaneous pressure recordings, and 3) appropriate target branch(es) leading to the septal myocardium were responsible for intra-LV obstruction. ASA was performed as previously described [17].

### Statistical analysis

Data were expressed as a mean ± the standard deviation. We performed all statistical analyses with IBM SPSS statistics version 21 and compared continuous variables between HNCM and LVOT obstruction (LVOTO) or segments with and without LGE using a Student's t-test or the Mann-Whitney's U test when appropriate. We compared categorical variables using the chi-square test and assessed the relationships between the summed uptake score and clinical parameters using Pearson's correlation. Pre- and post-ASA values were compared using a paired t-test or the Wilcoxon signed-rank test. Statistical significance was determined when a P-value was less than 0.05.

## Results

### Participants

Thirty HCM patients underwent ^18^F-FDG-PET/CT (age 64.4±10.5 years, 14 male [46.6%]). Twelve patients had HNCM, 14 had HOCM with LVOTO, two had HOCM with isolated mid-ventricular obstruction (MVO), and two had DHCM. The clinical characteristics of the HNCM and LVOTO patients in our study are listed in Table 1 and that of the 30 HCM patients in S1 Table. NYHA functional class and BNP values were significantly higher in HOCM patients with LVOTO than HNCM patients (2.5±0.6 vs. 1.6±0.9, *P = 0*.*016*, and 406.3±335.3 vs. 176.0±196.6 pg/ml, *P = 0*.*043*, respectively). E/e’ of HOCM patients with LVOTO was significantly higher than that of HNCM patients (25.1±13.6 vs. 14.2±4.8, *P = 0*.*040* at septal side and 19.3±11.5 vs. 8.8±2.8, *P = 0*.*030* at lateral side). Intra-LV pressure gradients of HOCM patients with LVOTO were significantly higher than that of HNCM patients (72.0±42.5 vs. 15.7±12.9 mmHg, *P<0*.*001*).

**Table 1:** Clinical characteristics of the study population.

	HNCM	LVOTO	*P-value*
			(HNCM vs. HOCM)
	(n = 12)	(n = 14)	
Age (years)	61.5±8.7	67.6±9.7	0.11
Male	5(41.7)	6 (42.8)	0.63
NYHA	1.6±0.9	2.5±0.6	0.016
BNP (pg/ml)	176.0±196.6	406.3±335.3	0.043
HSTnT (ng/ml)	0.02±0.01	0.03±0.04	0.40
Hypertension	7 (58.3)	9 (64.2)	0.76
Dyslipidemia	7 (58.3)	11 (78.6)	0.27
Diabetes mellitus	2 (16.7)	0 (0)	0.11
Smoking	4 (33.3)	2 (14.2)	0.25
eGFR (mL/min/1.73m^2^)	64.5±7.5	55.6±16.5	0.07
Syncope	0 (0)	4 (28.6)	0.04
Family history	4 (33.3)	3 (21.4)	0.50
AF	1 (8.3)	4 (28.6)	0.19
VT/VF	1 (8.3)	1 (7.1)	0.91
Medication			
β-blocker	12 (100)	14 (100)	1.00
CCB	6 (50)	6 (42.9)	0.51
Class Ia	5 (41.7)	12 (85.7)	0.025
ICD/ pacemaker	1 (8.3)	1 (7.1)	0.72
TTE			
IVST (mm)	14.3±2.7	14.9±4.1	0.89
LVPWT (mm)	8.9±1.7	11.3±4.0	0.046
Max. LV wall thickness (mm)	15.9±2.0	16.6±3.0	0.53
LAD (mm)	39.2±6.3	43.7±8.2	0.12
E/A	1.1±0.5	1.2±0.6	0.69
E/e’ (sep)	14.2±4.8	25.1±13.6	0.040
E/e’ (lat)	8.8±2.8	19.3±11.5	0.030
Intra-LVPG (mmHg)	15.7±12.9	72.0±42.5	<0.001
SAM	4 (33.3)	9 (64.2)	0.12
^18^F-FDG-PET/CT			
Summed uptake score	11.6±10.8	22.0±16.3	0.064
Extent score	5.2±3.8	7.3± 4.5	0.14
Mean SUV (LV)	1.5±0.4	2.0± 1.1	0.18
Maximum SUV (LV)	3.4±2.0	4.3± 2.8	0.35
CMR imaging			
Performed	9 (75)	14 (100)	0.084
performed with gadolinium enhancement	9 (75)	13 (92.9)	0.24
LV mass index (g/cm2)	78.6±21.2	100.5±39.9	0.11
LVEF (%)	55.8±10.5	61.7±8.0	0.17
The presence of LGE	8 (66.7)	7(50)	0.46
LVOT jet	4(33.3)	13 (92.9)	0.007
MR jet	1 (8.3)	10 (71.4)	0.001

Data are expressed as mean ± standard deviation or number of the patients (percentage). P-value compares HNCM and LVOTO for continuous variables using Student's t-test and categorical and ordinal variables using chi-square test. P-value compares between two groups for NYHA using Mann-Whitney's U test. HNCM: hypertrophic non-obstructive cardiomyopathy, LVOTO: left ventricular outflow tract obstruction, NYHA: New York Heart Association functional class, BNP: brain natriuretic peptide, HSTnT: high-sensitive troponin T, Smoking: previous or current smoker, eGFR: estimated glomerular filtration rate, Syncope: past history of syncope, Family history: family history of HCM, AF: atrial fibrillation, VT: ventricular tachycardia, VF: ventricular fibrillation, CCB: calcium channel blocker, Class Ia: class Ia antiarrhythmic agents, TTE: transthoracic echocardiography, IVST: interventricular septum thickness, LVPWT: left ventricular posterior wall thickness, LAD: left atrium demension, E/A: peak early diastolic LV filling velocity/peak atrial filling velocity ratio, E/e’ (sep) and E/e’ (lat): the ratio between standard Doppler derived transmitral early diastolic velocity (E) and pulsed Doppler derived early diastolic velocity of the mitral annulus (e’) measured at the septal site and at the lateral site of the mitral annulus, Intra-LVPG: intra-left ventricular pressure gradient, SAM: systolic anterior motion of the mitral valve, ^18^F-FDG-PET/CT: myocardial ^18^F-fluorodeoxyglucose positron emission tomography/computed tomography, SUV: standardized uptake value, CMR: cardiac magnetic resonance, LV mass Index: left ventricular mass index, LVEF: left ventricular ejection fraction, LGE: late gadolinium enhancement, LVOT: left ventricular outflow tract, MR: mitral regurgitation.

### ^18^F-FDG and CMR analyses

**Fig 1:**
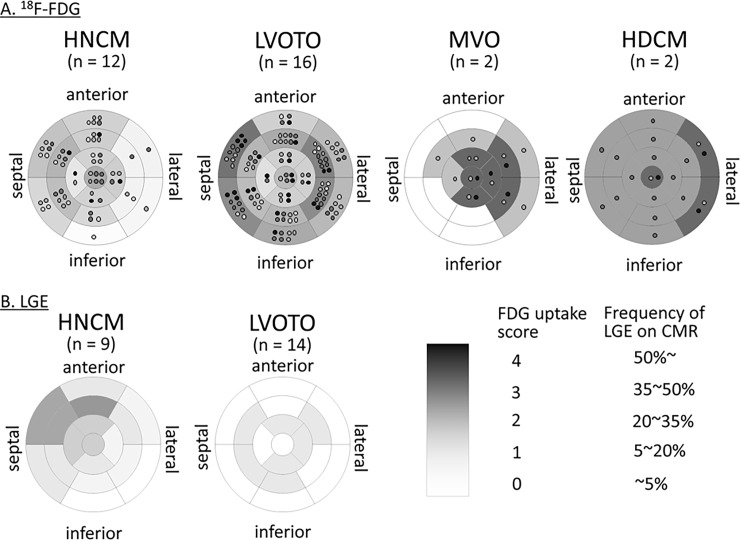
The degree of 18F-fluorodeoxyglucose (18F-FDG) uptake and the frequency of late gadolinium enhancement (LGE) at each LV 17 segment. (A) The 18F-FDG uptake in each type of HCM. The degree of 18F-FDG uptake was visually evaluated and semi-quantitatively scored using an uptake score (0: no uptake, 1: slight uptake, 2: mild uptake 3: moderate uptake, and 4: dense uptake). Light gray dot indicates the individual with slight uptake, middle gray dot mild uptake, dark gray dot moderate uptake, and black dot dense uptake. The mean uptake score was calculated in each segment and expressed by the density of gray color. (B) The frequency of LGE in HNCM and HOCM with LVOTO. The frequency of LGE was calculated in each LV segment and expressed by the density of gray color. The data of MVO and DHCM are not shown because only one patient per type received CMR.

The ^18^F-FDG uptake in each type of HCM is shown in Fig 1A. Patients with HNCM had ^18^F-FDG uptake mostly in the basal and mid-anteroseptal segments, corresponding to a hypertrophied myocardium. On the other hand, HOCM patients with LVOTO had strong ^18^F-FDG uptake not only in the basal septal segments but also in the mid-lateral segments. HOCM patients with isolated MVO had ^18^F-FDG uptake mainly in the segments close to the apical or mid-free wall. The detailed degree of ^18^F-FDG uptake in each segment is shown in S2 Table and S1 Fig.

The frequency of LGE in each 17 segment in the HNCM and LVOTO types of HOCM is shown in Fig 1B. In HNCM patients, LGE was frequently observed in the anteroseptal segments. On the other hand, LGE was randomly observed in HOCM with LVOTO. The segments with LGE had a higher ^18^F-FDG uptake score compared to those without (1.4 ± 1.3 vs. 0.7 ± 1.2, P = 0.02) in HNCM patients, whereas the difference was not significant (2.0 ± 1.5 vs. 1.7 ± 1.5, P = 0.29) in HOCM patents. The frequency of LGE on each segment is shown in S3 Table.

**Fig 2:**
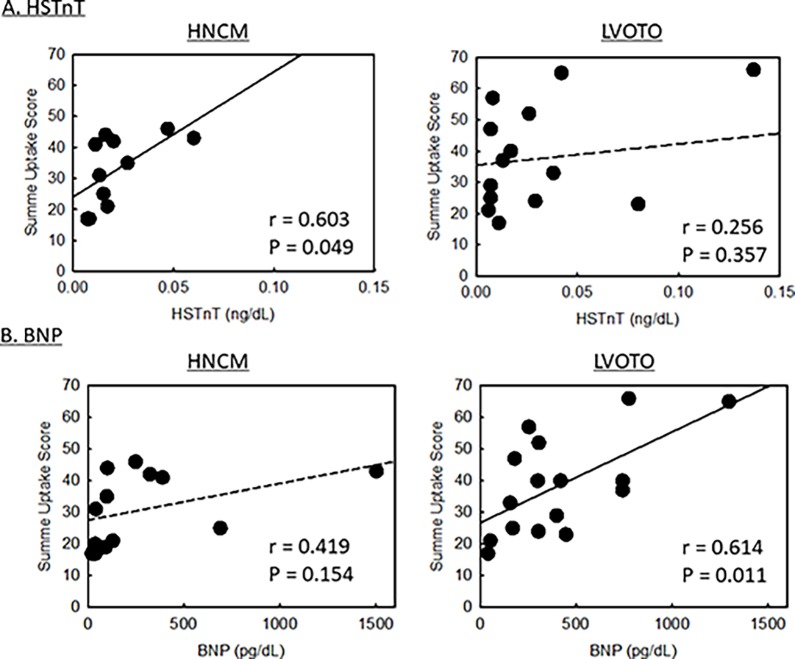
The relationships between summed uptake score of ^18^F-FDG and clinical parameters. (A) The relationships between the summed uptake score and high-sensitive troponin T (HSTnT) level in HNCM patients (the left panel) and in HOCM patients (the right panel). (B) The relationships between the summed uptake score and brain natriuretic peptide (BNP) level in HNCM patients (the left panel) and in HOCM patients (the right panel).

The relationships between the summed uptake score of ^18^F-FDG and HSTnT and BNP are shown in Fig 2. In HNCM patients, there was a positive correlation between the uptake score and HSTnT level (r = 0.603, *P = 0*.*049*) but not between the uptake score and BNP level (r = 0.419, *P = 0*.*154*). On the other hand, in HOCM patients, there as a positive correlation between the uptake score and BNP level (r = 0.614, *P = 0*.*011*) but not between the uptake score and HSTnT level (r = 0.256, *P = 0*.*357*).

### ASA procedure

**Fig 3:**
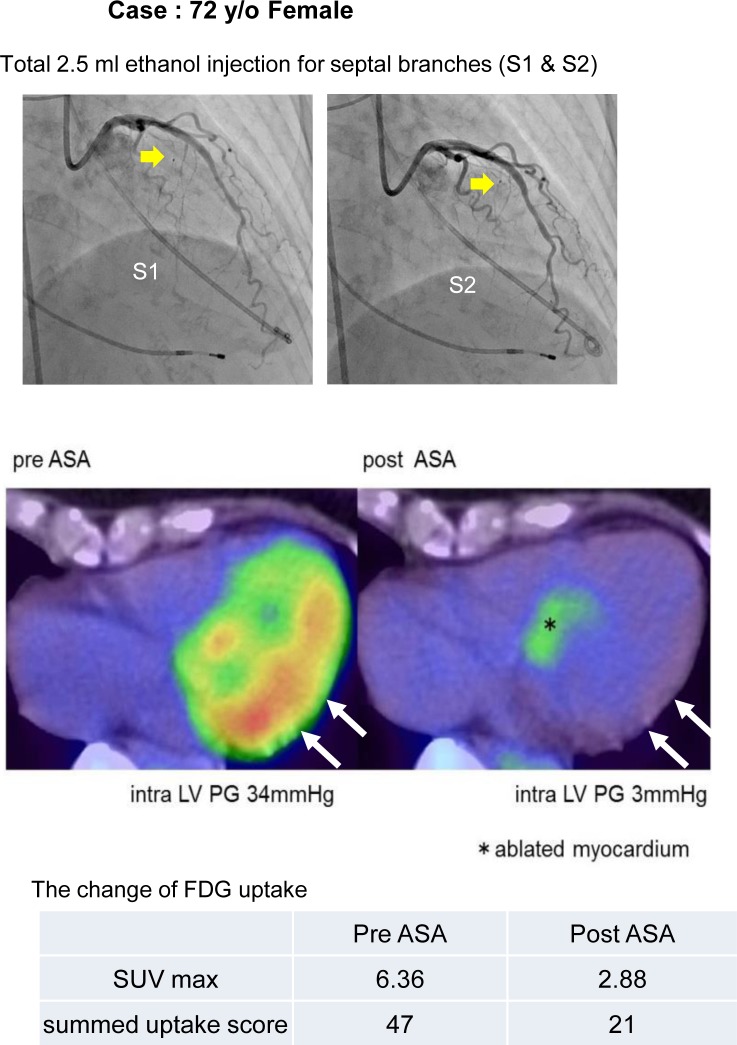
A representative case of ASA. Images of serial ^18^F-FDG-positron emission tomography (PET)/computed tomography (CT) in a representative case of an HOCM patient who received alcohol septal ablation therapy (ASA). Ablated septal branches are indicated with yellow arrows in the upper panels. In the middle panels, long axis trans-vertical LV views of ^18^F-FDG-PET /CT before and after ASA are shown. An ablated myocardium is indicated with a star symbol, and the portion with a significant reduction of ^18^F-FDG uptake in the lateral and posterior LV wall is indicated with white arrows. In the lower table, the changes of the maximum standardized uptake value (SUV max) and summed uptake score are shown.

Ten HOCM cases received ASA. A representative case of a 72-year-old woman with HOCM who received ASA is described in Fig 3. As well as the significant reduction of the intra-LV pressure gradient, ^18^F-FDG uptake in the lateral LV wall was attenuated (Fig 3).

**Fig 4:**
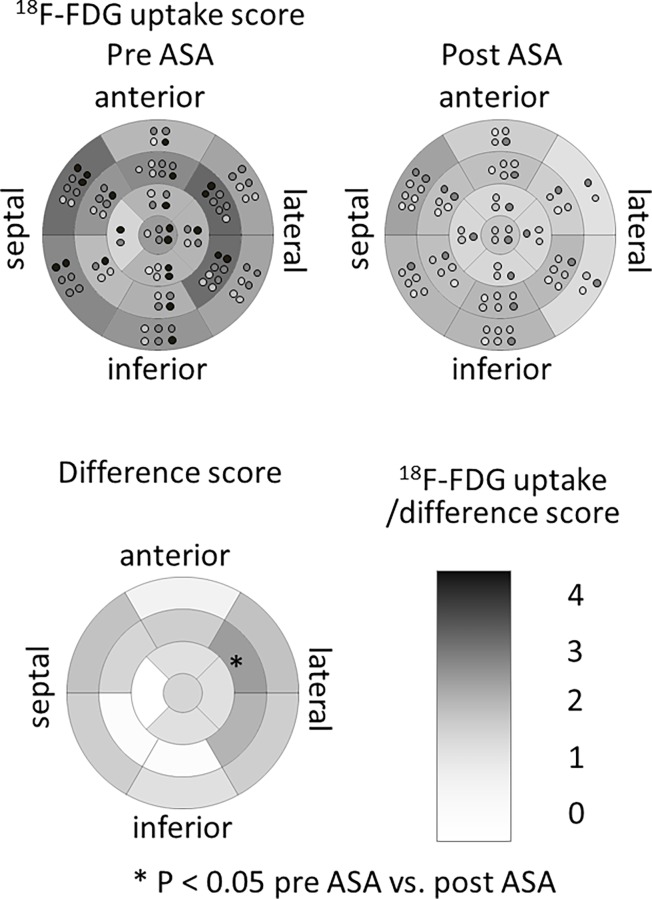
Change of 18F-FDG uptake score after ASA. Mean uptake score of ^18^F-FDG before and after ASA and difference score at each LV 17-segment. The mean uptake score was calculated with a semi-quantitative scoring method for each segment. Light gray dot indicates the individual with slight uptake, middle gray dot mild uptake, dark gray dot moderate uptake, and black dot dense uptake. The difference score was calculated by subtracting the post-value from the pre-value for each segment. The values were expressed by the density of gray color. * P<0.05 between pre- and post-ASA uptake score.

Table 2 shows the details of the procedures and the changes of clinical parameters after ASA. ASA reduced the intra-LV pressure gradient from 60.2±39.9 to 21.7±22.7 mmHg (*P<0*.*001*) and improved NYHA functional class from 2.5±0.5 to 1.1±0.3 (*P<0*.*001*). As well as the improvement of several clinical parameters such as BNP (from 496.1±361.7 to 238.2±155.5, *P = 0*.*004*) and E/e’ at both septal and lateral sides (from 25.1±9.7 to 19.1±5.9, P = 0.010, and from 17.6±8.6 to 9.8±3.1, *P = 0*.*022*, respectively), the summed uptake score of ^18^F-FDG uptake significantly reduced (from 26.9±14.5 to 15.6±16.1, *P = 0*.*022*). The semi-quantitative analysis of ^18^F-FDG uptake in the LV myocardium, expressed as maximum SUV of the entire LV, was significantly reduced (from 5.6±2.6 to 3.2±2.1, *P = 0*.*040*). The reduction was particularly significant in the lateral region (from 5.5±2.6 to 2.9±2.2, *P = 0*.*024*), not in the anteroseptal or inferior regions (from 4.5±2.6 to 2.9±1.6, *P = 0*.*12*, or from 4.4±2.9 to 2.5±1.5, *P = 0*.*085*, respectively). The semi-quantitative scoring method also revealed the significant reduction of ^18^F-FDG uptake in the mid-anterolateral segment after ASA (Fig 4). The detailed ^18^F-FDG uptake score before and after ASA and difference of uptake score at each segment are shown in S4 Table and S2 Fig.

**Table 2:** Changes of clinical parameters after ASA therapy.

HOCM patients (n = 10)			Pre-ASA	Post-ASA	P-value
Age (years)			64.7±12.6		-
Male			4 (40)		-
ASA procedure					
The amount of injected alcohol (ml)			3.3±1.3		-
The number of treated septal branches			2.0±0.7		-
Peak CPK (IU/L)			1740.5±635.7		-
Peak CPK-MB (IU/L)			257.1±122.0		-
NYHA functional class			2.5±0.5	1.1±0.3	<0.001
BNP (pg/ml)			496.1±361.7	238.2±155.5	0.004
HSTnT (ng/ml)			0.01±0.03	0.03±0.04	0.64
TTE					
IVST (mm)			15.6±4.3	13.1±2.7	0.072
LVPWT (mm)			11.5±4.3	11.3±4.4	0.38
LAD (mm)			46.4±7.5	40.2±7.8	0.004
E/A			1.3±0.5	1.1±0.7	0.16
E/e' (sep)			25.1±9.7	19.1±5.9	0.010
E/e' (lat)			17.6±8.6	9.8±3.1	0.022
Intra-LVPG (mmHg)			60.2±39.9	21.7±22.7	<0.001
SAM			5 (50)	2 (20)	0.17
Intra-LVPG by catheter measurement (mmHg)			81.0±63.0	26.6±31.2	0.002
^18^F-FDG-PET/CT imaging					
Summed uptake score			26.9±14.5	15.6±16.1	0.022
Extent score			8.1±3.9	6.0±3.5	0.26
Mean SUV		Entire LV	2.5±1.1	1.7±0.8	0.077
Anteroseptal			2.3±1.2	1.7±0.6	0.14
Inferior			2.5±1.1	1.6±0.8	0.062
Lateral			2.6±1.3	1.6±0.9	0.053
Maximum SUV		Entire LV	5.6±2.6	3.2±2.1	0.040
Anteroseptal			4.5±2.6	2.9±1.6	0.12
Inferior			4.4±2.9	2.5±1.5	0.085
Lateral			5.5±2.6	2.9±2.2	0.024
CMR imaging					
LV mass index (g/cm2)			102.3±43.9	101.8±51.4	0.47
LVEF (%)			59.7±7.3	60.4±10.2	0.56
Presence of LGE			5 (50)	5 (50)	0.67
LVOT jet			10 (100)	7 (70)	0.10
MR jet			7 (70)	3 (30)	0.086

Data are expressed as mean ± standard deviation or number of the patients (percentage). P-value compares pre- and post-ASA for continuous variables using paired Student's t-test and for categorical variables by using chi-square test.

HOCM: obstructive hypertrophic cardiomyopathy, ASA: alcohol septal ablation, CPK: creatine phosphokinase, CPK-MB: creatine phosphokinase MB isoenzyme, NYHA: New York Heart Association, BNP: brain natriuretic peptide, TTE: transthoracic echocardiography, IVST: interventricular septum thickening, LVPWT: left ventricular posterior wall thickening, LAD: left atrium dimension, E/A: peak early diastolic LV filling velocity/peak atrial filling velocity ratio, E/e’ (sep) and E/e’ (lat): the ratio between standard Doppler-derived transmitral early diastolic velocity (E) and pulsed Doppler-derived early diastolic velocity of the mitral annulus (e’) measured at the septal site and at the lateral site of the mitral annulus, intra-LVPG: intra-LV pressure gradient SAM: systolic anterior motion of the mitral valve, ^18^F-FDG-PET/CT: myocardial ^18^F-fluorodeoxyglucose positron emission tomography/computed tomography, SUV: standardized uptake value, CMR: cardiac magnetic resonance, LV mass index: left ventricular mass index, LVEF: left ventricular ejection fraction, LGE: late gadolinium enhancement, LVOT: left ventricular outflow tract, MR: mitral regurgitation.

## Discussion

In the present study, we addressed regional myocardial ^18^F-FDG uptake which indicate energy metabolic shift or inflammatory response using ^18^F-FDG-PET/CT in each type of HCM. Uptake of ^18^F-FDG was limited in a hypertrophied myocardium in HNCM whereas extensive ^18^F-FDG uptake beyond the hypertrophied myocardium was observed in HOCM. The extent and degree of ^18^F-FDG uptake was closely related with the HSTnT level, as well as the parameters of diastolic LV function and BNP. Reduction of intra-LV obstruction using ASA can affect myocardial metabolic shift in the lateral myocardium of HOCM.

### Methodological considerations

In order to more precisely evaluate myocardial metabolism, we contrived a new carbohydrate restriction diet protocol in addition to conventional ^18^F-FDG-PET. A major obstacle in diagnosing myocardial metabolism by using ^18^F-FDG-PET is the high physiological accumulation of ^18^F-FDG in the myocardium, which interferes with the recognition of abnormal ^18^F-FDG uptake [14]. Suppression of this unfavorable uptake is important to identify myocardium metabolism status. It is reported that carbohydrate restriction over 24 hours significantly suppresses physiological accumulation of ^18^F-FDG in the myocardium [14]. Therefore, all patients underwent over 24 hours carbohydrate restriction (glucose, <10g) before ^18^F-FDG-PET/CT study.

In addition to the semi-quantitative scoring method, we also measure SUV which is considered to be more objective evaluation. The summed uptake score was positively correlated with mean SUV of entire LV (R = 0.673, P<0.001, S3 Fig). This indicates the appropriateness of our uptake scoring evaluation and sufficient restriction of physiological ^18^F-FDG uptake to other organ.

### The significance of ^18^F-FDG uptake in HCM

The energy source of a normal myocardium is mainly fatty acids (over 90%)[10]. In some pathologic conditions such as ischemia or inflammation, the energy source of the myocardium shifts to glucose metabolism from fatty acids[8]. ^18^F-FDG is an analogue of glucose, and ^18^F-FDG-PET is used to visualize glucose metabolism in vivo [18]. In HCM, we postulate that the following four mechanisms are involved, in which glucose metabolism is necessary: 1) increased energy demand due to myocardial hypertrophy[19], 2) inflammatory response caused by inflammatory cell infiltration[20], 3) myocardial ischemia due to microangiopathy [21, 22], and 4) demand myocardial ischemia due to supply/demand mismatch of blood flow [23]. We speculate that the accumulation of ^18^F-FDG that we observed at non-hypertrophied lesions in LVOTO patients mainly involves the demand myocardial ischemia, because blood supply decreases due to increased extravascular compressive forces and oxygen demand increases in order to contract against wall stress under the increased pressure overload condition.

The usefulness of ^18^F-FDG-PET in HCM has been reported in previous studies [24–26]. Uehara reported that ^18^F-FDG uptake is increased not only in hypertrophied but also in the non-hypertrophied myocardium in HCM with asymmetrical septal hypertrophy or a dilated phase of HCM, whereas it was limited in a hypertrophied myocardium in HNCM [26]. The present study, in agreement with Uehara’s report, identified extensive pathophysiological metabolism beyond the hypertrophied myocardium in HOCM using ^18^F-FDG-PET/CT although the PET protocol was different from that in the previous study. As novel findings, we demonstrated that abnormal metabolism at a non-hypertrophied myocardium can be reversed by the attenuation of intra-LV obstruction.

We found that^18^F-FDG uptake score was positively correlated with HSTnT level in HNCM patients. Because HSTnT is generally considered as the cardiac marker of ongoing myocardial injury, ^18^F-FDG uptake observed in hypertrophic myocardium may be reflecting inflammatory response or ischemia caused by microcirculation disturbance. On the other hand, we found the positive correlation between ^18^F-FDG uptake score and BNP level in HOCM patients, indicating that ^8^F-FDG uptake was more extensively observed in failing heart in this condition. Flow disturbance at LVOT due to obstruction causes metabolic abnormality in not only hypertrophied but also remote myocardium possibly due to demand ischemia, as well as increased secretion of BNP.

### LGE and ^18^F-FDG uptake

Since LGE seen on CMR is known to reflect myocardial fibrosis, the existence of LGE in HCM indicates an advanced stage of this disease [27]. The pathohistological changes of HCM are characterized by myocyte disarray, enlarged cardiomyocytes, and deposition of interstitial fibrosis [27]. To develop dense myocardial fibrosis, detected as LGE on CMR, sustained pathological stress is considered to be necessary. In this process, microvascular ischemia or inflammatory response plays an important role. A previous study has reported a significant relationship between microvascular ischemia and myocardial fibrosis [28]. In the present study, by comparing CMR and ^18^F-FDG-PET/CT, we clarified the spatial relationship between LGE and ^18^F-FDG uptake in each type of HCM. In patients with HNCM, we found LGE as well as ^18^F-FDG uptake mostly in hypertrophied segments. This may indicate that the myocardium requiring glucose metabolism also has fibrotic changes. Therefore, it is possible that the ^18^F-FDG uptake may indicate the risk of the development of fibrosis in HNCM patients. On the other hand, in patients with LVOTO, ^18^F-FDG was widely distributed in both hypertrophied septal and remote lateral segments whereas LGE was rarely observed in those segments. The possible explanation of less frequent LGE segments compared with ^18^F-FDG uptake segments in LVOTO is that LGE reflects progressed myocardial cell damage, whereas ^18^F-FDG uptake reflects metabolic disorders which emerge from the early stage of myocardial cell damage. Since the pathological condition responsible for their symptom in patients with LVOTO who were referred for ASA was mainly due to mechanical intra LV obstruction, LVOTO patients enrolled in the present study might be still on the way to the development of myocardial fibrosis.

### Serial assessment of ^18^F-FDG-PET/CT before and after ASA

ASA is an optional treatment for symptomatic HOCM patients who are refractory to medical therapy [29]. Increasing evidence has supported the beneficial effect of ASA in improving symptoms associated with HOCM [30, 31]. Timmer et al. have reported that ASA has favorable effects on myocardial metabolism. ^15^O-H_2_O PET and ^11^C-acetate PET showed the changes of microvascular function and myocardial metabolism by relief of LVOTO [32]. In the present study, we found altered metabolism or myocardial inflammation detected as ^18^F-FDG uptake which observed in a non-hypertrophied myocardium can be reversed by ASA. We attributed this improvement to attenuation of demand myocardial ischemia, which is associated with wall stress from pressure overload. A previous study has reported the beneficial effect of ASA on the systolic movement of lateral (remote) wall using CMR. In the present study, we demonstrated the disappearance of ^18^F-FDG uptake in the remote myocardium as well as diastolic function in the lateral side. The metabolic improvement may explain, at least in part, the mechanism of the improvement of systolic function observed in the previous study [33].

Currently, indication for ASA is fundamentally determined by the degree of pressure gradient and assessment of life-limiting symptoms, assessed by NYHA grade [30, 31]. However, a discrepancy between these two factors is frequently observed [17]. We therefore postulate that the severity and extent of ^18^F-FDG uptake may be helpful for determining the indication for ASA, although further study is necessary to confirm the generalizability of our findings. ^18^F-FDG-PET/CT can be also useful to evaluate the therapeutic efficacy of ASA.

### Limitations

The number of patients was small due to the limited capacity of PET imaging in our institution. Particularly, we only included two isolated MVO patients and two dilated HCM patients in this study. Therefore, we did not include their data in our analysis and avoided commenting on isolated MVO or dilated HCM, although data are shown in the figures and tables as references. Secondly, the study was basically cross-sectional and no long-term follow-up data were addressed. Thus, prognostic implication of ^18^F-FDG-PET/CT could not be assessed in this study. Finally, although we observed significant ^18^F-FDG uptake in hypertrophied myocardium in HNCM patients, it remains unclear whether the uptake is indeed pathophysiological or mostly due to the greater thickness of the hypertrophied septum. The increase of ^18^F-FDG uptake might be related to a relative, partial-volume dependent overestimation of the true ^18^F-FDG tissue concentration.

## Conclusion

We addressed regional myocardial energy metabolic shift using ^18^F-FDG-PET/CT in HNCM and HOCM patients. Uptake of ^18^F-FDG is limited in the hypertrophied myocardium in HNCM whereas extensive ^18^F-FDG uptake beyond the hypertrophied myocardium was observed in HOCM. The fact that ASA can contribute to the improvement of myocardial metabolism or inflammation in the remote myocardium suggests the novel, beneficial effect of ASA besides the symptomatic improvement.

## Supporting information

S1 Table The clinical characteristics of the 30 HCM patients. (DOCX)

S2 Table The score of ^18^F-fluorodeoxyglucose uptake at each segment. (DOCX)

S3 Table The frequency of late gadolinium enhancement at each segment. (DOCX)

S4 Table The uptake score of ^8^F-fluorodeoxyglucose before and after ASA and difference score at each segment. (DOCX)

S1 Fig The comparisons of ^18^F-fluorodeoxyglucose uptake score of between HNCM and LVOTO at each segment. (TIF)

S2 Fig The comparisons of ^8^F-fluorodeoxyglucose uptake score between pre and post PTSMA at each segment. (TIF)

S3 Fig Linear regression analysis of summed uptake score of ^8^F-fluorodeoxyglucose and mean SUV of entire LV. (TIF)
