# Supplementary material for: Evaluation of myocardial glucose metabolism in hypertrophic cardiomyopathy using 18F-fluorodeoxyglucose positron emission tomography
Source: PLoS One. 2017 Nov 27;12(11):e0188479. doi: 10.1371/journal.pone.0188479 (PMC5703458; doi:10.1371/journal.pone.0188479)
Supplement: S1 Table — (DOCX) [file pone.0188479.s001.docx]

S1 Table. Clinical characteristics of the study population

|  | HNCM | HOCM | | DHCM | *P-value* |
| --- | --- | --- | --- | --- | --- |
|  |  | LVOTO | Isolated MVO |  | (HNCM vs. HOCM) |
|  | (n=12) | (n=14) | (n=2) | (n=2) |  |
| Age (years) | 61.5±8.7 | 67.6±9.7 | 62 | 55.5 | 0.11 |
| Male | 5(41.7) | 6 (42.8) | 2 (100) | 1 (50) | 0.63 |
| NYHA | 1.6±0.9 | 2.5±0.6 | 1.75 | 3.0 | 0.016 |
| BNP (pg/ml) | 176.0±196.6 | 406.3±335.3 | 314.7 | 166.8 | 0.043 |
| HSTnT (ng/ml) | 0.02±0.01 | 0.03±0.04 | 0.038 | 0.02 | 0.40 |
| Hypertension | 7 (58.3) | 9 (64.2) | 2 (100) | 2 (100) | 0.76 |
| Dyslipidemia | 7 (58.3) | 11 (78.6) | 2 (100) | 2 (100) | 0.27 |
| Diabetes mellitus | 2 (16.7) | 0 (0) | 1 (50) | 0 (0) | 0.11 |
| Smoking | 4 (33.3) | 2 (14.2) | 0 (0) | 1 (50) | 0.25 |
| eGFR ( mL/min/1.73m^2^ ) | 64.5±7.5 | 55.6±16.5 | 56.7±16.1 | 58 | 0.07 |
| Syncope | 0 (0) | 4 (28.6) | 0 (0) | 1 (50) | 0.04 |
| Family history | 4 (33.3) | 3 (21.4) | 0 (0) | 1 (50) | 0.50 |
| AF | 1 (8.3) | 4 (28.6) | 1 (50) | 0 (0) | 0.19 |
| VT/VF | 1 (8.3) | 1 (7.1) | 0 (0) | 1 (50) | 0.91 |
| Medication |  |  |  |  |  |
| β-blocker | 12 (100) | 14 (100) | 2 (100) | 2 (100) | 1.00 |
| CCB | 6 (50) | 6 (42.9) | 2 (100) | 0 (0) | 0.51 |
| Class Ia | 5 (41.7) | 12 (85.7) | 1 (50) | 1 (50) | 0.025 |
| ICD/ pacemaker | 1 (8.3) | 1 (7.1) | 1 (50) | 0 (0) | 0.72 |
| TTE |  |  |  |  |  |
| IVST (mm) | 14.3±2.7 | 14.9±4.1 | 18.5 | 11.5 | 0.89 |
| LVPWT (mm) | 8.9±1.7 | 11.3±4.0 | 11.5 | 7.5 | 0.046 |
| Max. LV wall  thickness (mm) | 15.9±2.0 | 16.6±3.0 | 17.2±3.8 | 11.6 | 0.53 |
| LAD (mm) | 39.2±6.3 | 43.7±8.2 | 47.0 | 40 | 0.12 |
| E/A | 1.1±0.5 | 1.2±0.6 | 1.45 | 2.0 | 0.69 |
| E/e’ (sep) | 14.2±4.8 | 25.1±13.6 | 23.3 | 18.7 | 0.040 |
| E/e’ (lat) | 8.8±2.8 | 19.3±11.5 | 10.1 | 16.9 | 0.030 |
| Intra-LVPG  (mmHg) | 15.7±12.9 | 72.0±42.5 | 47.5 | - | <0.001 |
| SAM | 4 (33.3) | 9 (64.2) | 2 (100) | 0 (0) | 0.12 |
| ^18^F-FDG-PET/CT imaging | | | | | |
| Summed uptake score | 11.6±10.8 | 29.0±16.3 | 19.5 | 26.5 | 0.064 |
| Extent score | 5.2±3.8 | 7.3± 4.5 | 8.5 | 10 | 0.14 |
| CMR imaging |  |  |  |  |  |
| Performed | 9 (75) | 14 (100) | 1 (50) | 1 (50) | 0.084 |
| performed with gadolinium enhancement | 9 (75) | 13 (92.9) | 1 (50) | 1 (50) | 0.24 |
| LV mass index (g/cm2) | 78.6±21.2 | 100.5±39.9 | 125.3 | 61.4 | 0.11 |
| LVEF (%) | 55.8±10.5 | 61.7±8.0 | 56.6 | 36.4 | 0.17 |
| The presence of LGE | 8 (66.7) | 7(50) | 1 (50) | 1 (50) | 0.46 |
| LVOT jet | 4(33.3) | 13 (92.9) | 1 (50) | 0 (0) | 0.007 |
| MR jet | 1 (8.3) | 10 (71.4) | 0(0) | 0 (0) | 0.001 |

Data are expressed as mean ± standard deviation or number of the patients (percentage). P-value compares HNCM and LVOTO for continuous variables using Student's t-test and categorical and ordinal variables using chi-square test. P-value compares between two groups for NYHA using Mann-Whitney's U test. HNCM: hypertrophic non-obstructive cardiomyopathy, HOCM: hypertrophic obstructive cardiomyopathy, LVOTO: left ventricular outflow tract obstruction, MVO: mid ventricular obstruction, DHCM: dilated phase of HCM, NYHA: New York Heart Association functional class, BNP: brain natriuretic peptide, HSTnT: high-sensitive troponin T, Smoking: previous or current smoker, eGFR: estimated glomerular filtration rate, Syncope: past history of syncope, Family history: family history of HCM, AF: atrial fibrillation, VT: ventricular tachycardia, VF: ventricular fibrillation, CCB: calcium channel blocker, Class Ia: class Ia antiarrhythmic agents, TTE: transthoracic echocardiography, IVST: interventricular septum thickness, LVPWT: left ventricular posterior wall thickness, LAD: left atrium demension, E/A: peak early diastolic LV filling velocity/peak atrial filling velocity ratio, E/e’ (sep) and E/e’ (lat) : the ratio between standard Doppler derived transmitral early diastolic velocity (E) and pulsed Doppler derived early diastolic velocity of the mitral annulus (e’) measured at the septal site and at the lateral site of the mitral annulus, Intra-LVPG: intra-left ventricular pressure gradient, SAM: systolic anterior motion of the mitral valve, ^18^F-FDG-PET/CT: myocardial ^18^F-fluorodeoxyglucose positron emission tomography/computed tomography, CMR: cardiac magnetic resonance, LV mass Index: left ventricular mass index, LVEF: left ventricular ejection fraction, LGE: late gadolinium enhancement, LVOT: left ventricular outflow tract, MR: mitral regurgitation.
