# Supplementary material for: Evaluation of myocardial glucose metabolism in hypertrophic cardiomyopathy using 18F-fluorodeoxyglucose positron emission tomography
Source: PLoS One. 2017 Nov 27;12(11):e0188479. doi: 10.1371/journal.pone.0188479 (PMC5703458; doi:10.1371/journal.pone.0188479)
Supplement: S2 Table — (DOCX) [file pone.0188479.s002.docx]

S2 Table. The score of ^18^F-fluorodeoxyglucose uptake at each segment

|  | All (n=30) | HNCM (n=12) | HOCM | | | DHCM (n=2) | P-value (HNCM vs. LVOTO) |
| --- | --- | --- | --- | --- | --- | --- | --- |
| Segment |  |  | All (n=16) | LVOTO type (n=14) | MVO type (n=2) |  |  |
| 1 | 0.8±1.3 | 0.8±1.1 | 0.7±1.4 | 0.8±1.4 | 0 | 1.5 | 0.69 |
| 2 | 1.6±1.5 | 1.0±1.2 | 2.1±1.6 | 2.4±1.5 | 0 | 1.5 | 0.025 |
| 3 | 1.2±1.5 | 0.7±0.9 | 1.6±1.7 | 1.9±1.7 | 0 | 1.5 | 0.068 |
| 4 | 0.7±1.3 | 0.1±0.3 | 1.2±1.5 | 1.4±1.5 | 0 | 1.5 | 0.015 |
| 5 | 0.8±1.2 | 0.2±0.6 | 1.1±1.3 | 1.1±1.3 | 1 | 2.5 | 0.023 |
| 6 | 0.8±1.2 | 0.3±0.9 | 1.1±1.3 | 1.1±1.3 | 1 | 2.5 | 0.037 |
| 7 | 1.2±1.4 | 0.9±1.3 | 1.4±1.4 | 1.4±1.5 | 1 | 1.5 | 0.33 |
| 8 | 1.3±1.4 | 1.3±1.5 | 1.3±1.4 | 1.3±1.5 | 1 | 1.5 | 0.96 |
| 9 | 1.1±1.4 | 1.1±1.2 | 1.1±1.5 | 1.2±1.5 | 0 | 1.5 | 0.91 |
| 10 | 0.9±1.3 | 0.6±1.2 | 1.1±1.4 | 1.3±1.4 | 0 | 1.5 | 0.13 |
| 11 | 1.3±1.4 | 0.3±0.6 | 2.0±1.4 | 1.9±1.4 | 2.5 | 1.5 | 0.001 |
| 12 | 1.3±1.5 | 0.3±0.9 | 2.1±1.4 | 2.0±1.5 | 2.5 | 1.5 | <0.001 |
| 13 | 0.9±1.3 | 0.8±1.1 | 1.0±1.4 | 0.5±1.3 | 2 | 1.5 | 0.81 |
| 14 | 0.6±1.3 | 0.5±1.2 | 0.6±1.3 | 0.5±1.3 | 1 | 1.5 | 0.91 |
| 15 | 1.0±1.5 | 0.8±1.4 | 1.0±1.5 | 0.9±1.5 | 2.5 | 1.5 | 0.84 |
| 16 | 1.0±1.4 | 0.9±1.4 | 1.0±1.5 | 0.9±1.5 | 2.5 | 1.5 | 0.87 |
| 17 | 1.4±1.5 | 1.3±1.4 | 1.3±1.4 | 1.1±1.7 | 2.5 | 2.5 | 0.72 |

The scores of FDG uptake are expressed as follows; 0: no uptake, 1: slight uptake, 2:mild uptake 3: moderate uptake, and 4: dense uptake. Data are expressed as mean ± standard deviation. P-value compares HNCM and HOCM using Mann-Whitney's U test.

HNCM: non-obstructive hypertrophic cardiomyopathy, HOCM: obstructive hypertrophic cardiomyopathy, LVOTO: left ventricular outflow tract obstruction, MVO: mid ventricular obstruction, DHCM: dilated phase of HCM, FDG: fluorodeoxyglucose
