# Supplementary material for: Evaluation of myocardial glucose metabolism in hypertrophic cardiomyopathy using 18F-fluorodeoxyglucose positron emission tomography
Source: PLoS One. 2017 Nov 27;12(11):e0188479. doi: 10.1371/journal.pone.0188479 (PMC5703458; doi:10.1371/journal.pone.0188479)
Supplement: S3 Table — (DOCX) [file pone.0188479.s003.docx]

S3 Table. The frequency of late gadolinium enhancement at each segment

| Segment | HNCM (n=9) | HOCM | | | DHCM (n=1) | P-value (HNCM vs. LVOTO) |
| --- | --- | --- | --- | --- | --- | --- |
|  |  | All (n=14) | LVOTO type (n=13) | MVO type (n=1) |  |  |
| 1 | 2 (22.2) | 0 | 0 | 0 | 1 | 0.16 |
| 2 | 4 (44.4) | 1 (7.1) | 1 (7.7) | 0 | 1 | 0.13 |
| 3 | 2 (22.2) | 1 (7.1) | 1 (7.7) | 0 | 1 | 0.36 |
| 4 | 0 | 0 | 0 | 0 | 1 | - |
| 5 | 0 | 0 | 0 | 0 | 0 | - |
| 6 | 1 (11.1) | 0 | 0 | 0 | 0 | 0.41 |
| 7 | 5 (55.5) | 1 (7.1) | 0 | 1 | 1 | 0.004 |
| 8 | 4 (44.4) | 2 (14.3) | 1 (7.7) | 1 | 1 | 0.066 |
| 9 | 2 (22.2) | 2 (14.3) | 2 (15.4) | 0 | 1 | 0.55 |
| 10 | 1 (11.1) | 2 (14.3) | 1 (7.7) | 1 | 0 | 0.66 |
| 11 | 1 (11.1) | 1 (7.1) | 1 (7.7) | 0 | 0 | 0.66 |
| 12 | 2 (22.2) | 2 (14.3) | 1 (7.7) | 1 | 1 | 0.36 |
| 13 | 3 (33.3) | 2 (14.3) | 1 (7.7) | 1 | 1 | 0.17 |
| 14 | 3 (33.3) | 1 (7.1) | 0 | 1 | 0 | 0.054 |
| 15 | 3 (33.3) | 2 (14.3) | 1 (7.7) | 1 | 0 | 0.17 |
| 16 | 2 (22.2) | 3 (21.4) | 2 (15.4) | 1 | 0 | 0.55 |
| 17 | 3 (33.3) | 1 (7.1) | 1 (7.7) | 0 | 0 | 0.17 |

Data are expressed as number of the patients (percentage) who have late gadolinium enhancement on each segment. P-value compares two groups using chi-square test.

HNCM: non-obstructive hypertrophic cardiomyopathy, HOCM: obstructive hypertrophic cardiomyopathy, LVOTO: left ventricular outflow tract obstruction, MVO: mid ventricular obstruction, DHCM: dilated phase of HCM
