# Supplementary material for: Evaluation of myocardial glucose metabolism in hypertrophic cardiomyopathy using 18F-fluorodeoxyglucose positron emission tomography
Source: PLoS One. 2017 Nov 27;12(11):e0188479. doi: 10.1371/journal.pone.0188479 (PMC5703458; doi:10.1371/journal.pone.0188479)
Supplement: S4 Table — (DOCX) [file pone.0188479.s004.docx]

S4 Table. The uptake score of ^18^F-FDG before and after ASA and difference score at each segment

|  | ^18^F-FDG uptake score | | Difference score | P-value  (pre-ASA vs. post-ASA) |
| --- | --- | --- | --- | --- |
| Segment | Pre-ASA | Post-ASA |  |  |
| 1 | 1.1±1.6 | 0.8±1.1 | 0.3±1.4 | 0.52* |
| 2 | 2.5±1.5 | 1.5±1.1 | 1.0±1.7 | 0.095* |
| 3 | 2.0±1.8 | 1.2±1.1 | 0.8±1.9 | 0.23* |
| 4 | 1.7±1.6 | 1.2±1.1 | 0.5±1.5 | 0.32* |
| 5 | 1.5±1.4 | 0.6±1.1 | 0.9±1.6 | 0.11* |
| 6 | 1.5±1.4 | 0.5±1.1 | 1.0±1.6 | 0.084* |
| 7 | 1.9±1.4 | 1.0±1.2 | 0.9±1.6 | 0.11* |
| 8 | 1.7±1.6 | 1.0±1.2 | 0.7±1.6 | 0.21* |
| 9 | 1.1±1.5 | 1.0±1.2 | 0.1±1.6 | 0.84* |
| 10 | 1.3±1.6 | 1.2±1.1 | 0.1±1.4 | 0.83* |
| 11 | 2.3±1.4 | 1.1±1.1 | 1.2±1.8 | 0.058* |
| 12 | 2.4±1.4 | 0.8±1.1 | 1.6±2.0 | 0.032* |
| 13 | 2.2±1.6 | 1.7±1.2 | 0.5±1.1 | 0.50^#^ |
| 14 | 0.7±1.5 | 0.7±1.2 | 0.0±0.9 | 1^#^ |
| 15 | 1.2±1.7 | 0.7±1.2 | 0.5±1.4 | 0.50^#^ |
| 16 | 1.2±1.6 | 0.7±1.2 | 0.5±1.7 | 0.50^#^ |
| 17 | 1.6±1.8 | 0.9±1.2 | 0.7±1.8 | 0.25^#^ |

The scores of FDG uptake are expressed as follows; 0: no uptake, 1: slight uptake, 2: mild uptake 3: moderate uptake, and 4: dense uptake.

Difference score was calculated by the subtraction of post-ASA value from pre-ASA value. P-value compares pre- and post-ASA values using paired Student's t-test* or Wilcoxon Signed Rank Test^#^. ^18^F-FDG: ^18^F-fluorodeoxyglucose, ASA: alcohol septal ablation
