# Supplementary figures and images for: Evaluation of myocardial glucose metabolism in hypertrophic cardiomyopathy using 18F-fluorodeoxyglucose positron emission tomography
Source: PLoS One. 2017 Nov 27;12(11):e0188479. doi: 10.1371/journal.pone.0188479 (PMC5703458; doi:10.1371/journal.pone.0188479)

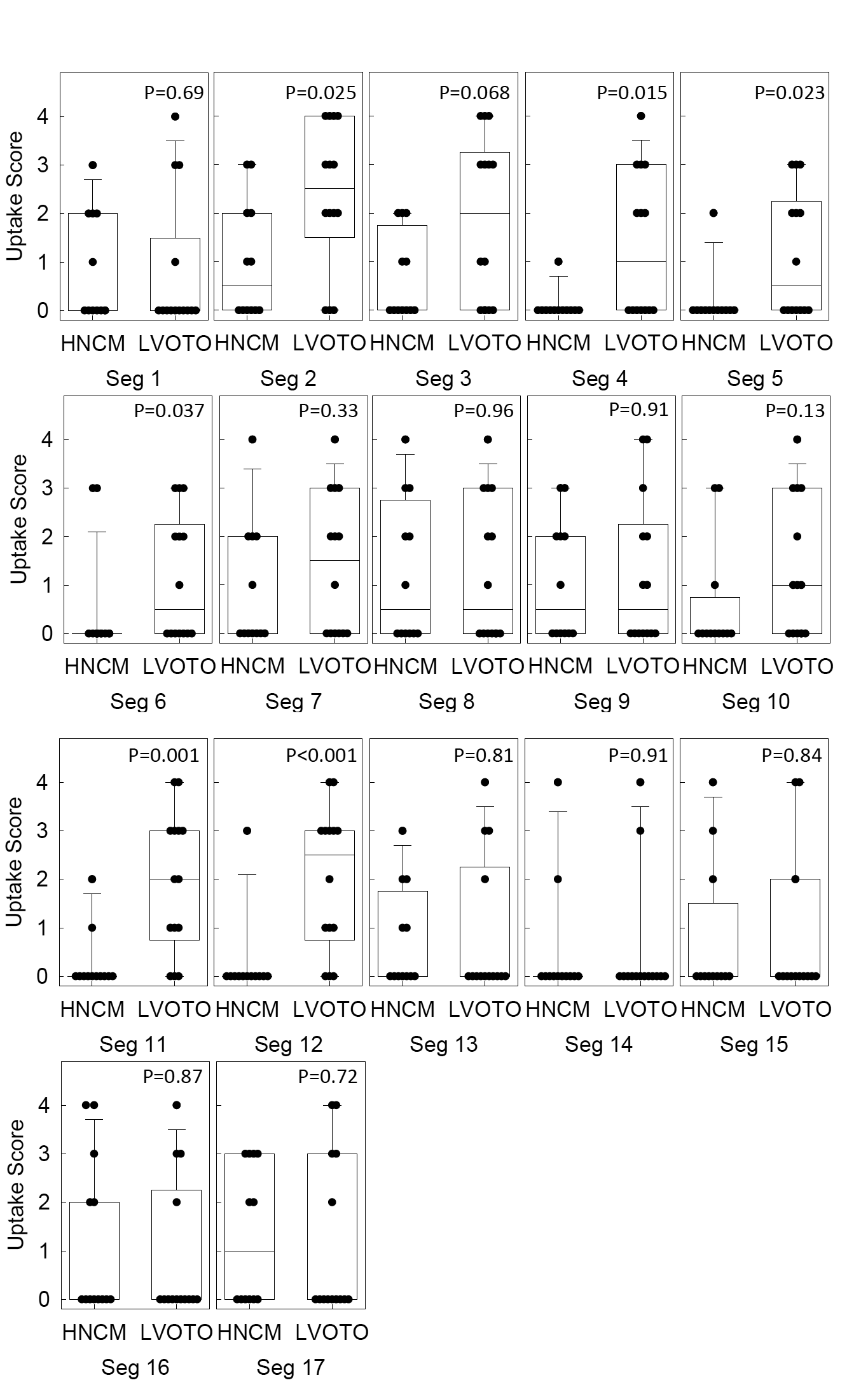

Supplement: S1 Fig — (TIF) [file pone.0188479.s005.tif]

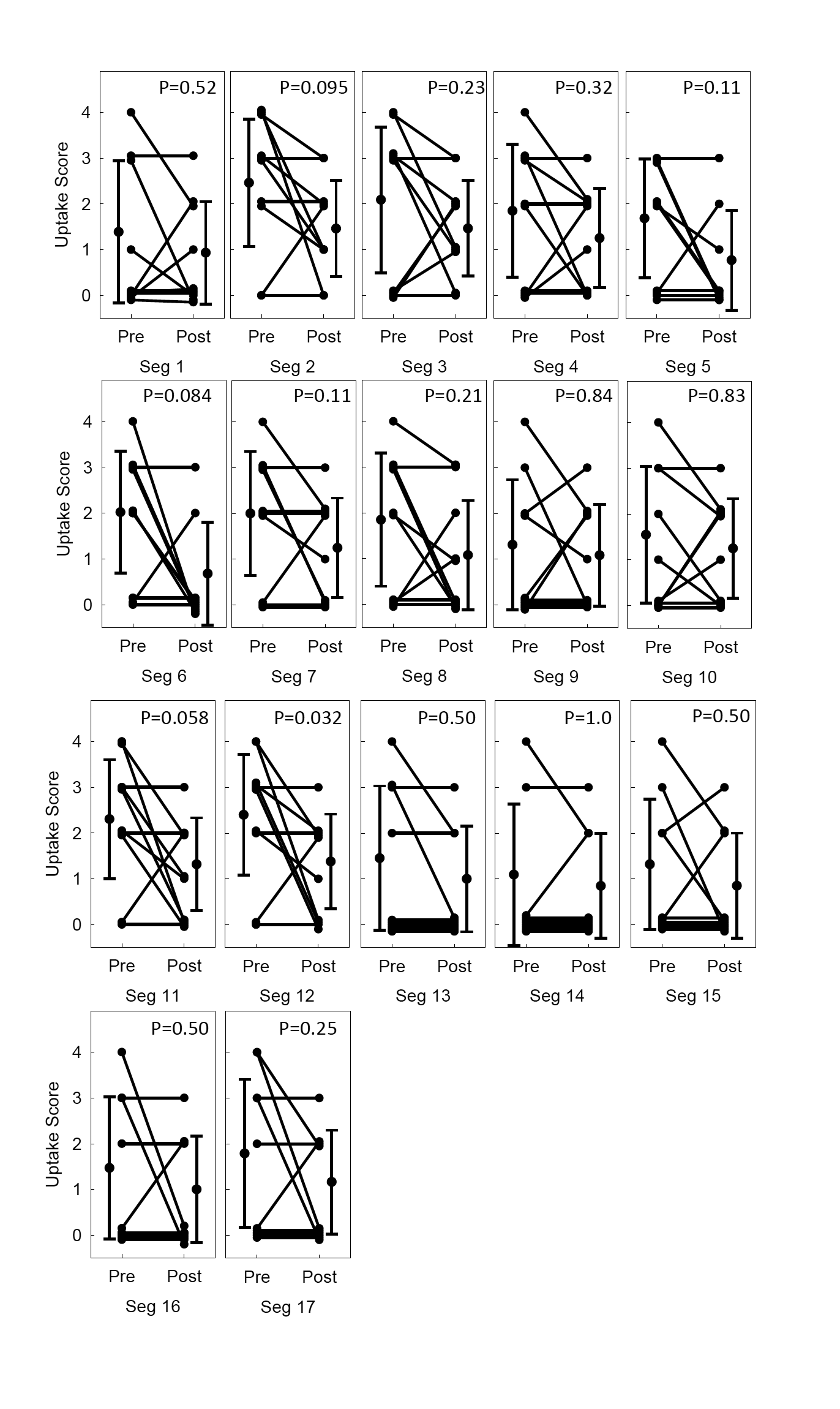

Supplement: S2 Fig — (TIF) [file pone.0188479.s006.tif]
